# Identification of differential gene expression in in vitro FSH treated pig granulosa cells using suppression subtractive hybridization

**DOI:** 10.1186/1477-7827-4-35

**Published:** 2006-07-07

**Authors:** A Bonnet, PO Frappart, P Dehais, G Tosser-Klopp, F Hatey

**Affiliations:** 1 INRA laboratoire de Génétique cellulaire BP52627 chemin de borde rouge 31326 Castanet cedex, France; 2 Department of Genetic St. Jude Children's Research Hospital 332N.Lauderdale Street, Memphis TN 38105, USA

## Abstract

FSH, which binds to specific receptors on granulosa cells in mammals, plays a key role in folliculogenesis. Its biological activity involves stimulation of intercellular communication and upregulation of steroidogenesis, but the entire spectrum of the genes regulated by FSH has yet to be fully characterized.

In order to find new regulated transcripts, however rare, we have used a Suppression Subtractive Hybridization approach (SSH) on pig granulosa cells in primary culture treated or not with FSH. Two SSH libraries were generated and 76 clones were sequenced after selection by differential screening. Sixty four different sequences were identified, including 3 novel sequences. Experiments demonstrated the presence of 25 regulated transcripts.

A gene ontology analysis of these 25 genes revealed (1) catalytic; (2) transport; (3) signal transducer; (4) binding; (5) anti-oxidant and (6) structural activities. These findings may deepen our understanding of FSH's effects. Particularly, they suggest that FSH is involved in the modulation of peroxidase activity and remodelling of chromatin.

## Background

The development of ovarian follicles leading to ovulation requires endocrine regulation by the gonadotropins FSH and LH as the main actors. The complex regulatory network also includes steroids and peptides (e.g. growth factors, inhibins) acting via the autocrine and paracrine pathways. Recent studies have highlighted the importance of FSH in ovarian follicle maturation: in FSH-deficient mice the folliculogenesis is blocked prior to antral formation [1,2]. In order to obtain functional gametes, granulosa cell (GC) communication with the oocyte also seems essential [2,3]. GCs constitute an important compartment in the mammalian ovarian follicle, contributing to follicle development. They actively participate in the endocrine function of the ovaries by secreting oestradiol or progesterone under FSH stimulation. Besides their functional importance, GCs have been intensively studied for their convenient isolation. In murine, porcine, and bovine species they constitute a well-standardized model for the *in vitro *study of GC function, including hormonal regulation.

Even if much data has been accumulated on the action of gonadotropins on GCs, the entire spectrum of genes regulated by FSH is not known. Besides recent advances in the generation of normalized cDNA libraries [4,5] and expression analysis with differential display PCR and microarrays [6,7], SSH approach [8] was more efficient in accessing low-level expressed transcripts. We have therefore used the SSH method to isolate FSH-regulated genes in pig primary GC. These results increase our understanding of the physiological processes involved in the response of GC to FSH. In particular, FSH may play a role in the modulation of peroxidase activities and the remodelling of chromatin.

## Methods

### Cell cultures

Pig granulosa cells were isolated and cultured as described previously by Hatey *et al *[9]. Briefly, the granulosa cells were isolated from medium (around 3 mm in diameter) healthy follicles from immature swine ovaries. Cells were plated and grown to confluence in a serum-containing medium, which was replaced after 5 days of culture by a serum-free medium with or without FSH (Gonal-F™ 0.5 UI/ml, Serono laboratory) and incubated for a 48-h period before RNA extraction. FSH stimulation efficiency was tested both by measuring progesterone secretion in the culture medium using HPLC analysis [10] and by controlling the increase in P450scc and IGF1 genes expression using Northern blot analysis.

### RNA and polyA extraction

Total RNA extraction was performed according to Chomczynski and Sacchi [11] with minor modifications [12]. Poly(A)-containing RNA was selected with Dynabeads mRNA Purification Kit (Dynal) following the manufacturer's instructions. For quality control, total RNA and mRNA were denatured with formaldehyde and size-fractionated through a 1% agarose gel according to standard methods. The integrity of each RNA sample was checked by ethidium bromide staining of the gel. Before reverse transcription, DNase I treatment of RNA was performed as described [13].

### Reverse-Transcription

Total DNase I treated RNA (2 μg) from control and induced cells were used for reverse transcription (RT) using Superscript™ II Reverse Transcriptase (Invitrogen) and oligo-dT15 primers (Roche) according to the manufacturer's recommendations.

### Suppression subtractive hybridization (SSH)

SSH was performed with 1 μg of mRNA using the Clontech PCR-Select cDNA Subtraction Kit (Clontech) with minor modifications. The primary PCR amplification was achieved through 30 PCR cycles starting with 1 μl of 6 fold diluted second hybridization reaction. The secondary PCR amplification was achieved through 12 PCR cycles starting with 2 μl of 10 fold diluted primary PCR amplification. The PCR products were cloned using the TOPO™ TA Cloning^R ^Kit (Invitrogen). Agarose gel analysis allowed the exclusion of empty clones or those containing more than one product. Forward subtraction H1 (FSH/C) used mRNA of control cells as a driver to select genes induced by FSH and reverse subtraction H2 (C/FSH) used mRNA of FSH-induced cells as a driver to select genes repressed by FSH.

### Differential screening

As the final SSH products were enriched, but not strictly composed of differentially expressed cDNAs, a screening procedure was set up to sort out the false positive clones. Inserts were amplified by PCR starting from each colony. Each cDNA was amplified by PCRusing Invitrogen Taq polymerase: initiation with one cycle of 7 min at 94°C and amplification with 20 cycles (94°C for 30 s, 68°C for 30 s and 72°C for 1.5 min). Primers used were nested PCR primer1 (5'-TCGAGCGGCCGCCCGGGCAGGT-3') and nested PCR primer2 (5'-AGGGCGTGGTGCGGAGGGCGGT-3'). PCR products were checked by electrophoresis on agarose gel. PCR products were denaturated in NaOH 0.5 M, EDTA 25 mM and 5% bromophenol blue, vacuum transferred onto two identical Hybond N membranes (Millipore) and UV crossed-linked. Macro-arrays were subsequently hybridized with the different probes (cDNA of control and treated cells). The screening was performed visually.

### Probe labelling and hybridization

Probe labelling with dCTP α ^32^P and hybridization of Northern-blot or macro-arrays were performed as described [9].

### Sequencing reaction and analysis

The inserts of regulated clones were amplified from the plasmid using Primer1 (5'-GTAATACGACTCACTAGGGC-3'), and Primer2 (5'-TGTAGCGTGAAGACGACAGAA-3'). PCR products were purified using Wizard^® ^PCR Preps DNA Purification System (Promega). Sequencing was performed using the ABI PRISM™ Big Dye™ Terminator Cycle Sequencing Ready Reaction Kit (Applied Biosystems) and the Perkin-Elmer 377 apparatus (Perkin-Elmer Cetus). Sequences were submitted to EMBL database and accession numbers are given in Table 2. Sequence assembling was done using the Wisconsin Genetic Computer Group software package (Wisconsin Package, version 10.0, Genetic Computer Group). Edited sequence data were analysed with the Advanced BLAST program of the EMBL web site for similarities with known genes or ESTs in the entire EMBL databases.

**Table 2:** Identification of cDNA clones. The table gives the identification of the cDNA clones using blast analysis against EMBL/NCBI database. The clones were ranked by HUGO symbol (Human Genome Organisation). H1-clones correspond to forward hybridization (FSH-induced genes) and H2-clones to reverse hybridization (FSH-repressed genes).

Clone			Matching Sequences					
clone	EMBL accession number	insert size (bp)	identification	species	Similarity		accession number	HUGO symbol

					%	bp		
H2-146	AJ704910	255	actin, alpha 2, smooth muscle, aorta	HS	96	211	MN_001613	ACTA2
H2-96	AJ704907	111	actin, gamma 1	HS	89	119	NM_001614	ACTG1
H1-2	AJ704867	186	cytosolic dihydrodiol dehydrogenase 3	BT	90	115	D49542	AKR1C3
H2-222	AJ704918	643	annexin A5	HS	93	495	CR541842	ANXA5
H1-378	AJ704899	76	ADP-ribosylation factor 1	HS	91	74	AF493881	ARF1
H1-98	AJ704876	166	mitochondrial ATPase 6	SS	98	165	AF250315	ATP6
H1-138	AJ704880	354	calpain I light subunit	SS	99	353	M11778	CAPNS1
H1-357	AJ704896	107	Cctg for chaperonin	HS	96	104	X74801	CCT3
H1-124	AJ704878	177	Pig complement cytolysis inhibitor	SS	99	171	M84639	clu
H1-61	AJ704870	245	alpha collagene human type IV(alport syndrome)	SS	82	162	M31115	col4A5
H2-108	AJ704909	410	cytochrome c oxidase II	SS	99	410	U18827	COXII
H1-253	AJ704887	99	chondroitin sulfate proteoglycan 6	HS	89	88	BC047324	CSPG6
H1-280	AJ704890	251	cytochrome P-450 (SCC)	SS	99	249	X13768	CYP11A1
H1-365	AJ704898	110	7-dehydrocholesterol reductase (DHCR7)	HS	95	111	AF062481	DHCR7
H1-140	AJ704881	143	farnesyl diphosphate synthase	BT	89	75	AF461050	FDPS
H1-185	AJ783757	366	glutathione peroxidase 3 (plasma) (GPX3)	HS	87	354	NM_002084	GPX3
H2-200	AJ704914	179	glycogenin	HS	83	179	NM_004130	GYG
H2-105	AJ704908	228	Homo sapiens heterochromatin protein 1, binding protein 3	HS	92	219	BC045660	HP1BP3
H1-1	AJ704866	256	3 β hydroxysteroidogenase	SS	99	256	AF232699	HSD3B1
H2-265	AJ704919	299	inositol polyphosphate-1-phosphatase	HS	89	229	NM_002194	INPP1
H2-175	AJ704912	187	matrin 3	HS	88	186	NM_018834	MATR3
H1-100	AJ704877	237	NAC alpha	HS	92	237	AY034001	NACA
H1-242	AJ704885	481	complete mitochondrial DNA	SS	100	450	AJ002189	NADH1
H2-201	AJ785755	170	Sus scrofa breed Landrace mitochondrion	SS	100	170	AF304202	NADH5
H1-33	AJ704869	141	peroxiredoxin 2	BT	86	117	AF305562	PDX2
H1-266	AJ704888	135	non-selenium glutathione phospholipid hydroperoxide peroxidase (phgpx gene)	SS	100	135	AJ243849	phgpx
H2-29	AJ704900	123	proteasome (prosome, macropain) 26S subunit, ATPase	HS	86	123	NM_002806	PSMC6
H1-358	AJ704897	87	cDNA DKFZp686F06244	HS	97	87	HSM806676	Q8NEW0
H1-73	AJ704872	120	ribosomal protein S15A	HS	93	130	BC001697	RPS15A
H1-129	AJ704879	467	Sterol regulatory element binding protein cleavage-activating protein (SCAP)	SS	88	486	AY705447	SCAP
H1-90	AJ704873	199	high density lipoprotein receptor SR-BI mRNA	SS	99	199	AF467889	SCARB1
H1-96	AJ704875	404	splicing factor 3a, subunit 3	HS	93	401	NM_006802	SF3A3
H1-62	AJ704871	90	solute carrier family 25	HS	85	98	BC008737	SLC25A6
H2-44	AJ704902	529	thrombospondin 1	BT	90	527	AB005287	THBS1
H1-94	AJ704874	114	tenascin	HS	91	114	D49542.1	TNC
H2-72	AJ704904	133	ubiquitin associated protein 2 (UBAP2), transcript variant 1	HS	91	106	NM_018449.2	UBAP2
H1-202	AJ704883	178	ubiquitin-conjugating enzyme E2E 2	HS	98	178	BC022332	UBE2E2
H1-305	AJ704893	178	angiogenic factor VG5Q,	HS	85	169	BC032844	VG5Q
H2-37	AJ704901	451	vimentin	HS	92	438	NM_003380	VIM
H2-170	AJ704911	191	breed Landrace mitochondrion	SS	100	190	AF304202	unknown
H2-202	AJ704915	120	domestica mitochondrial D-loop,	SS	100	120	AF276937	unknown
H1-180	AJ704882	178	885998 MARC 4PIG	SS	99	178	CF792994	unknown
H1-246	AJ704886	284	jns82_C06.f jns 5'	SS	99	284	CB481752	unknown
H1-276	AJ704889	247	MI-P-CP0-nvm-c-03-0-UI.s2 MI-P-CP0	SS	99	236	BQ603741	unknown
H1-294	AJ704891	131	855499 MARC 4PIG 5'	SS	98	118	CF787582	unknown
H1-345	AJ704895	90	820907 MARC 3PIG 3'	SS	10	89	CF359642	unknown
H2-92	AJ704905	497	jns96_H04.f jns	SS	99	492	CB482726	unknown
H2-199	AJ704913	236	903820 MARC 4PIG 3'	SS	99	236	CK450726	unknown
H1-338	AJ704894	97	, Similar to RIKEN cDNA 2410141M05 gene	HS	91	92	BC008630	unknown
H2-216	AJ704917	107	RIKEN full-length enriched library, product: ribosomal protein S17	MM	94	93	AK007992	unknown
H2-288	AJ704920	162	CT02029A1F04 Equine Articular Cartilage	EC	87	114	CX601260	unknown
H1-14	AJ704868	221	endogenous retrovirus PERV-MSL	SS	97	221	AF038600	
H1-302	AJ704892	108	None	SS				
H2-95	AJ704906	137	None	SS				
H2-206	AJ704916	658	None	SS				

### Comparative RT-PCR analysis

**Figure 1:**
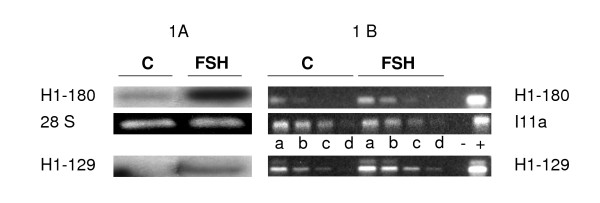
**validation of comparative RT-PCR**. Comparison ofNorthern and RT-PCR analysis with H1-180 and H1-129. A shows Northern analysis of total RNA (5 μg) from control (C) or FSH-treated GCs (FSH). Hybridization was performed with H1-180 and H1-129 cDNA probes coming from forward hybridization (H1 = FSH-induced genes). The amount and integrity of RNA in each lane was checked with ethidium bromide staining of the gels before transfer (28 S lane). B shows the results of PCR amplification of H1-180 and H1-129 cDNAs using specific primers. Total RNA was extracted from control (C) or FSH-treated (FSH) cells and was reverse-transcribed. For PCR, four different amounts of each cDNA were used: **a: **1 ng, **b: **250 pg, **c: **50 pg, **d: **10 pg and 2 controls (-: water, **+**: corresponding insert). PCR amplification of I11a (plant external control added to each RNA before RT) was performed on the same samples (control and FSH-treated cDNAs) to check efficiency of the RT and PCR processes.

The comparative RT-PCR was performed as described by Tosser-Klopp *et al *[13]. Briefly, 0.5 μCi of α ^32^P labelled dCTP (specific activity >3000 Ci.mmole^-1^, Perkin Elmer) was added to each reverse transcription to monitor the transcription. First strand cDNAs were purified by G50 chromatography and quantified by measuring the incorporated radioactivity. For each cDNA sample (control and FSH-treated cells) the same dilutions were made starting from the same amount of material. The PCR conditions were: denaturation 5 min at 94°C, followed by (22–40) cycles of (30 s, 94°C, 30 s, 56°C, 1.5 min, 72°C) in presence of 1.5 mM MgCl_2_. The primers are listed in table 4. Ten μl of PCR products were analysed by electrophoresis on 1.5% agarose gel. Each experiment was performed at least three times. An external standard (plant mRNA: I11a accession number: y10291) was added to each RNA sample (200 fg for 2 μg total RNA sample) before reverse transcription and allowed the control of the cDNAs quantifications and dilutions using specific primers (Figure 1).

**Table 4:** Localisation on pig chromosomes. This table gives the localisation obtained using SCHP and/or ImpRH panel hybrid. The result of SCHP consists in the chromosomal region with statistical scores: error risk and probability. For IMpRH the results were obtained by 2-point analysis. We indicate the number of hybrid clones used (90 or 118 Hybrids), the retention as a % frequency (% ret), the bearing chromosome, the closest marker, the location in Ray, and the load score value. We have indicated the chromosomal localisation in humans when known; the expected localisation in pigs derives from comparative mapping between humans and pigs.

Clone	Putative identification	Somatic localisation			IMpRH panel used		IMpRH localisation using two point analysis				Human localisation	pig expected localisation
		*Location*	*% Error risk*	*Prob.*		% ret	Chr	Name of the most linked marker	Location	LOD		

H1-2	AKR1C3				90	34	14	EST-AR023C05	4.74	4.81	10p 15 p 14	10
H1-61	col4A5	X p 24	0.10	0.77	90	50	X	SW1426	7.1	14.32	X q22	X
H1-129	SCAP				90	25	13	EST-AR062F01	11.44	19.28	3 p 21.31	13
H1-140	FDPS	4 p14-p15	0.10	1.00	90	24	4	IMpRH01700	26.17	15.94	1 q 22	4
H1-180	EST	9 p21-p24	5	0.63	118	35	9	SW911	3.83	10.89		
H1-246	EST	9 p21-p24	5	0.63	118	40	9	SW911	3.83	14.49		
H1-276	EST	10 p1-p16	0.10	0.80	118	29	2	PTH3	10.97	5.05		
H2-44	THBS1	1 p24-p25	1	0.81	118	29	1	348A10F	27.08	5.95	15 q 15	1
H2-92	EST	14?	0.50	1.00	90	31	14	RhC21D6F	19.69	15.71		
H2-95	EST	4 p14-p15	1	0.76	118	35	4	S0107	18.07	12.08		

### Regional assignment

Genomic localisation of the PCR products was done using the pig somatic cell hybrid panel (SCHP) as described previously by Tosser-Klopp *et al*. [13]. Each hybrid was scored for the presence of a pig specific DNA fragment and the assignments were performed using online software [14].

We also used the IMpRH panel 7000 rads [15] as described by Lahbib-Mansais *et al*.[16]. The results of radiation hybrid PCR products were analysed using the IMpRH mapping tool [17,18].

### Functional Gene Ontology annotation

The functional annotation analysis was performed on the 25 genes found to be regulated by Northern or comparative RT-PCR.

Since our sequences are rather short, and mainly belong to 3'UTR portions of genes, available public tools to assign GO molecular function to nucleic acid sequences lack sensitivity. So we set up the following workflow:

- Each sequence was compared to EMBL database via a NCBI blastn with an e-value limit set to 1e-3

- Blast results were parsed and filtered to keep hits having a) at least a coverage of 50% of the query sequence, b) 70% of identities, c) its coordinates inside a gene/mRNA/CDS location on the subject sequence.

- For each query sequence, candidate hits were sorted by a) their closeness to the pig genome, and b) their coverage and percent of identity.

- Annotations present in the EMBL record of the hit were parsed to look for cross references to proteins from UniProt/SwissProt databases

- Finally, using cross reference tables provided by GOA , molecular function GO was inferred from these best hits and associated to the sequences.

## Results

### Suppression subtractive hybridization

The SSH approach was performed on primary granulosa cells from cultures treated or not treated by FSH. mRNA from control granulosa cells was subtracted from the mRNA from FSH treated granulosa cells and *vice versa*. We isolated 226 clones from the forward hybridization H1 (FSH/C, FSH-induced genes) and 275 from the reverse subtraction H2 (C/FSH, FSH-repressed genes). In order to eliminate false positive clones, differential screening was used. The clones were spotted on two sets of macro-arrays and differential screening was performed using cDNA from the control and the treated cells as probes. We identified 28 (12.4%) induced cDNA clones and 19 (6.9%) repressed cDNA clones (Table 1). We also randomly selected 29 cDNA clones in both forward and reverse subtraction which provided no differential signals or no signal at all.

**Table 1:** Differential screening by macro-arrays. This table summarises the results of differential screening by SSH and the results of sequence analysis: Forward subtraction corresponds to H1 SSH and up-regulated genes by FSH (FSH/C). Reverse one corresponds to H2 SSH and down-regulated genes by FSH (C/FSH). Random selection corresponds to clones that provided no differential signals or no signal at all in both forward and reverse subtraction.

	Forward (FSH/C)	reverse (C/FSH)	random selection	total
number of clones	226	275		501
number of selected clones after screening	28 (12.4%)	19 (6.9%)	29	76
different clones	20	15	29	64
different genes	18	12	27	55

### Sequence analysis

These 76 selected clones (28+19+29) were sequenced and compared against public sequence databases. We identified 64 different sequences of which 3 were novel and 14 corresponded to different regions of the same 5 genes. We finally dealt with 55 genes. The results are summarized in Table 1. The complete list of genes is given in Table 2.

### Regulation study

Northern and comparative RT-PCR were used to analyse the differential expression of the selected genes. Out of the 55 genes, 18 could not be analysed according to their short length.

The results of the 37 genes left are presented in Table 3. Some genes could not be analysed by Northern (no signal or non interpretable results) and were thus analysed by RT-PCR. Also, 9 genes which gave a good signal by Northern analysis were also analysed by RT-PCR to compare the results (Fig 1).

**Table 3:** RNA expression. Results of RNA expression for the 37 genes. Nine genes are analysed by both Northern and comparative RT-PCR. For comparative RT-PCR the regulation was determined using cDNA dilutions from control cells and FSH treated cells as in Figure 1 and 2. In the Table, C = FSH corresponds to non regulated genes by FSH, FSH>C corresponds to up-regulated genes and C>FSH corresponds to down-regulated genes. NI corresponds to Non Interpretable, 0 corresponds to no signal. * these genes gave no signals during macro-array screening and thus were not controlled by Northern.

clones	putative identification	Northern	RT-PCR result	primers	Size of PCR product (pb)
H2-146	ACTA2	C>FSH			
H2-96	ACTG1	C>FSH			
H1-2	AKR1C3	FSH>C		U: GATCATCTCCAGCTGCTTGT L: AGGAGCTTCTGCCTAAGGAT	123
H2-222	ANXA5	C>FSH			
H1-98	ATP6	NI	C = FSH	U: TACGGCTAGGGCTACTGG L: TTCCGCCATAAAACCAAAAC	126
H1-138	CAPNS1	C = FSH	C = FSH	U: ACGGGGTGAGTCTAATGC L: CCAGTGAAGTGCCCAGAA	348
H1-357	CCT3	FSH>C			
H1-124	CLU	C = FSH	C = FSH	U: ACCCCAGCGTG CCTCT L: TTCCTCCGCCACAGTCTC	122
H1-61	Col4A5	FSH>C	FSH>C	U: CATAAAAAGCACAGGGAAAAG L: TTTCTCTTCTGCTGTATTCG	172
H2-108	COX II	FSH>C			
H1-280	CYP11A1	NI	FSH>C	U: CAAGTCATTCACGAGGTATC L: AGGAGATGCTGCGGGAG	161
H1-185	GPX3	FSH>C			
H2-105	HP1BP3	*	FSH>C	U: CTCAAGTCCATTTCCCAGC L: TCTGGCTCCTTGCGTGCT	114
H1-1	HSD3B1	FSH>C			
H2-265	INPP1	C>FSH			
H2-175	MATR3	*	C>FSH	U: GTTTTTGTTTTATCAGAATGG L: CTTATCTTTCTTAGTGGTGC	91
H1-100	NACA	C = FSH	C = FSH	U: AAG AGG AGA GTG AAG AGG A L: TTGAAACACCAAAAAAAGGGT	197
H1-242	NADH1	0	C = FSH	U: TTGGTGAATAGTTTTAGGGC L: CGAAAGGACAAGAGAAATGG	360
H1-33	PDX2	NI	C = FSH	U: TGACCCAGGAAAGCCAGA L: CTATGTCGCTCCAGGAAA	129
H1-14	porcine endogenous retrovirus perv-MSL	0	FSH>C	U: AAAACAGCAAAAAACCTAACC L: GCCCGTCTAACAAGAAAGC	180
H1-73	RP515A	C = FSH			
H1-129	SCAP	FSH>C	FSH>C	U: CCAGCCCAGATCCAGTG L: TAAACTACGGGGACCTGT	250
H1-90	SCARB1	NI	FSH>C	U: CAAGAAGCAAGACTGTAGG L: TGGCAACGGGAGGTGAG	160
H1-62	SLC25A6	NI	C = FSH	U: GTTCTCTTTTGCACAGCCG L: TTTTTTTGTGTCCTGATTTTATT	86
H2-44	THBS1	C>FSH		U: CTTTTCGTCTCCCTGGAAAT L: TGAAACTGATGGGCAAATCT	529
H1-94	TNC	0	C>FSH	U: GCTCCGTGGTGGACCTG L: CCCTCCACCTTCAGCTTG	104
H1-305	VG5Q	0	C = FSH	U: CCAGCATAAGCATCATTTTC L: AACAGAGAACCACCCTCCT	174
H2-37	VIM	0	C = FSH	U: GCTCAAGGGGACCAACGA L: TGGAAGAGGCAGAGAAATC	253
H2-95	New	0	C>FSH	U: AGTCACCAATCTTATCTCCA L: ATGAAACAATAGTCCAGGAAG	119
H1-202	unknown	C = FSH	C = FSH	U: ATCCTCAGTCAAAAGTGGCA L: CAATCAGAATCACTGTGCGT	154
H2-288	unknown	*	C = FSH	U: AAGGTGTGTTGATGTATTTTA L: TACAGGAAGGGAAGCATC	77
H1-294	unknown	FSH>C	FSH>C	U: CTTAGTTTCAGACTGGAGTT L: TTTAGCGGCTTGTTCACTCA	104
H2-92	unknown	*	FSH>C	U: ATGCCAACATCATCACCTCT L: CCCTCTAAACTGGGATCCAT	351
H2-170	unknown	FSH>C	FSH>C	U: AGACTTGTAGGTAGAGGTGA L: TAGGGTTTTTGGGGTATTTTT	99
H1-180	unknown	FSH>C	FSH>C	U: AAA TTG TAG GTA TAT GTG TCA L: TAGAGAATAAAATGGTCAGTAA	123
H1-246	unknown	FSH>C		U: CAATTCACCATCATCCACAA L: CCCAGCATTTCATACTGACC	284
H1-276	unknown	FSH>C		U: ACGATTCCTCTACTGAAAGCTG L: TGTTTTTGGCAAACAGCAG	247

**Figure 2:**
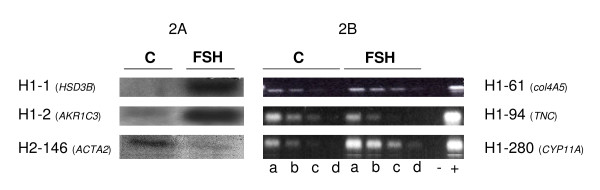
**Regulation studies**. Regulation by FSH of 6 genesusing Northern or comparative RT-PCR. H1 corresponds to forward hybridization (FSH-induced genes) and H2 to reverse hybridization (FSH-repressed genes). A: Northern analysis. B: comparative RT-PCR. Same conditions as in Figure 1.

Finally, out of the 37 genes, 25 were FSH-regulated (Table 3). In agreement with literature, *HSD3B1 *and *CYP11A *expression was induced by FSH in granulosa cells and *ACTA2 *expression was repressed by FSH (Fig 2).

FSH regulation of the expression of some other genes (*AKR1C3*, *col4A5*, *TNC*) is illustrated in Fig 2.

### Chromosomal localisation

Among the same 37 genes, 11 had already been located on pig chromosomes. Using the SCHP and the IMpRH hybrid panel, and the same primer pairs as for regulation studies, we tried to localise the 26 genes left. We successfully localised 10 genes, for the other 16 genes, the primers used amplified rodent DNA and stopped the analysis of the results (Table 4).

### Functional gene ontology analysis

The 25 regulated genes were analysed using gene ontology annotation in order to document the processes involved in FSH effect on granulosa cells from medium sized follicles.

**Figure 3:**
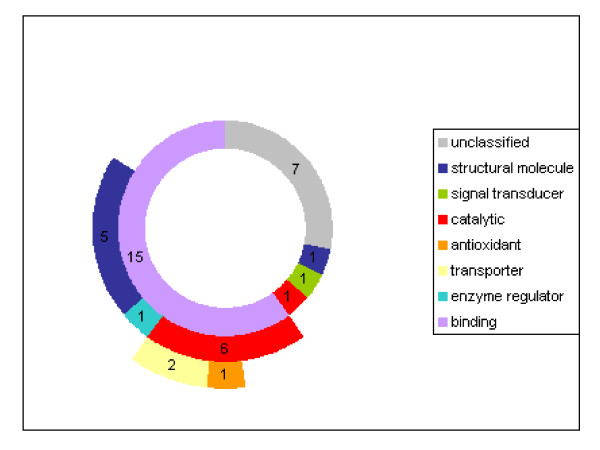
**Functional annotation: Molecular function**. The donut chart shows the distribution of the 25 regulated genes according to their molecular function (inner circle). Genes that can be assigned to more than one molecular function are indicated by outer partial rings in the donut chart. Numbers in this chart represent the number of sequences for each category.

For each sequence, we tried to assign molecular function GO identifiers using the methodology explained previously. We limited the analysis to the first levels of the molecular function ontology tree, just counting genes present in each node. The donut chart of figure 3 shows the distribution of the regulated genes according to their molecular function (see Additional file: supplementaldata-fig 3 for the data used to draw the chart). As genes can be assigned to more than one molecular function, some categories are drawn as outer partial rings in the donut chart (see also supplemental data). Numbers in this chart represent the number of genes observed for each category.

## Discussion

Our objective has been to identify the genes regulated in granulosa cells in response to FSH stimulation. The identification of such genes will give valuable information about the molecular processes associated with follicular growth. An increasing number of studies are being undertaken on candidate genes that regulate folliculogenesis, e.g. IGF [18], EGF [19], TGF-β [20] and other members of the transforming growth factor superfamily, like BMPs [21,22] and GDF-9 [23,24]. In parallel with such individual studies, we now have access to technologies that allow us to study a large number of genes simultaneously. We applied the SSH technique coupled with a differential screening procedure, to GCs treated with FSH compared with untreated controls. Among 501 clones initially obtained, 76 were sequenced and were found to correspond to 55 different genes (Table 2). Sequence analysis revealed redundancy from 2 different mechanisms: 1) gene redundancy, and 2) clone redundancy. This phenomenon is observed for highly expressed genes like- *HSD3B1 *and *alpha-actin*. High level of expression can also explain why two genes (*alpha-actin *and *complement cytosolic inhibitor*) are present in both the forward and reverse libraries (data not shown): the subtraction process may not be efficient for them.

### Regulation studies

Thirty seven genes were analysed for regulation. Ten genes did not give a signal with Northern blot or macroarrays (table 3) and were successfully analysed by RT-PCR, underlining the low level of expression of some genes identified through SSH: they probably correspond to rare transcripts.

*The Cox2 *gene and H2-170 gave discordant results when using subtraction or Northern analysis, possibly due to the high abundance of their mRNAs [25,26]. However, Northern experiments demonstrated clearly their high expression and regulation in granulosa cells.

We found some genes already known to be regulated by FSH in pigs e.g. *HSD3B *and *CYP11A *which are upregulated by FSH (Figure 2). We corroborated in pigs that *alpha *and *gamma actin *were downregulated by FSH, whereas *vimentin *was not affected, as observed in rats [27]. These genes validate the biological model and the analytical methods, as well as reinforcing previous studies [28].

### Localisation of cDNA and comparative mapping

This paper describes 10 localisations of genes or ESTs on the pig genome using the somatic or IMpRH hybrid panel. Three genes and 4 ESTs were assigned with a LOD score of >10. Localisations of these genes were in accordance with the data from the human genome. In spite of a relatively low LOD score of 5.95 (significance limit 6), *THBS1 *was assigned on chromosome 1 in agreement with the expected localisation, using a comparative map between humans and pigs [29,30].

The aim of these localisations was to identify candidate genes for reproductive Quantitative Trait Loci (QTL). This could be the case for col4A5 which is located on chromosome X at the same place as a plasma FSH concentration QTL [31]. Col4A5 was up regulated by FSH and is a positional candidate for this QTL. This result obviously deserves further investigation.

### Functional gene ontology analysis

In order to understand the pathways involved in the GC response to FSH, GO was used to cluster regulated genes according to their molecular function. Most of the tools used for automatic GO annotation of EST sequences, such as GOst [32] or OntoBlast [33], are mainly based on homology searches against well annotated protein databases. In our case, the sequence information obtained from the SSH clones is relatively short, and is biased towards 3' UTR; thus these tools are not convenient. Some other tools, such as Goblet [34], GoFigure [35] or Blast2GO [36] allow homology searches against nucleic databases, but once again with low sensitivity for short sequences. We thus used an in-house procedure to browse the GO classification and extract valuable information. The resulting classification (Fig 3 and Additional file) brings to light mainly 5 functional activities: "catalytic" and "signal transducer" activities directly linked with the steroid activity then "binding", "antioxidant" and "structural molecule" activities predominantly linked with differentiation pathways. On the other hand, our data demonstrate for the first time the involvement and/or the regulation of different genes in:

#### Catalytic activities

*COX-2 *was upregulated by FSH in our study and has been implicated as an important factor in female fertility [37]. In response to FSH, *COX-2 *induction could then stimulate progesterone production via the PGE2/EP2 pathway and play a role in ovulation by supporting cumulus expansion [38,39]. Indeed, mice deficient for *COX-2 *failed to ovulate, showing that *COX-2 *is necessary for ovulation [40].

#### Binding activity

Our data underline the FSH regulation of 4 genes involved in 1) DNA binding (*HP1-BP74*) 2) nucleic acid binding (*CCT3*), 3) lipid binding (*annexin V*), and 4) metal ion binding (*matrin*).

1) The upregulation of the *HP1-BP74 *gene (histone h1/h5 family), has never been shown before to be involved in granulosa cells development. Histones are highly conserved proteins involved in the package of chromosomes by interacting with DNA. Particularly, the HP1 family plays an important role in chromosomal biology and gene silencing. The *HP1-BP74 *gene was also shown to be involved in neuronal functional maturation [41]. This finding suggests an important role of H2-105 in GC differerentiation.

2) This study shows also the FSH up regulation of the *CCT3 *gene (*chaperonin containing TCP1*) known to mediate the folding of alpha- and beta-tubulin. FSH could thus intensify the remodelling of the microtubule cytoskeleton. Such a modification has been shown to be related to the maintenance and remodelling of heterochromatin during mammalian spermiogenesis [42].

3) We demonstrated the downregulation of *annexin V*: its binding to the cell membrane corresponds to the earlier events of apoptosis and is used to detect healthy live cells (annexin V negative) [43,44]. It is also a protein kinase C inhibitor which plays a potential role in cellular signal transduction [45]. This downregulation by FSH in GC may prevent apoptosis.

4) Finally, we found the downregulation of *matrin *by FSH. This protein that is localized in the nuclear matrix may have a role in RNA transcription thanks to its acidic region [46] and could be phosphorylated by nuclear PKC epsilon [47].

#### Signal transducer activity

Among the genes involved in signal transducer activity, our study showed the upregulation by FSH of the *Scavenger Receptor Class B Type I (SCARB1)*. This receptor is involved in both cholesterol delivery for steroid hormone production and in the recognition of apoptotic granulosa cells. We underline here the relationship with steroid hormone production, according to the downregulation of *annexin V *by FSH that suggests a differentiation process rather than an atretic one [48].

#### Anti-oxidant activities

During follicle growth, swine granulosa cells are physiologically exposed to a progressive oxygen shortage. In vitro reactive oxygen species (ROS), such as hydrogen peroxide (H2O2) and lipid hydroperoxide (LOOHs) provided by oxido-reduction reactions, can either negatively or positively affect the differentiation of the gametes notably during spermatogenesis [49,50]. Among the anti-oxidant activities, we noticed the upregulation by FSH of genes like glutathione peroxydase 3 (*GPX3*). *GPX3 *seems to regulate free hydoxyperoxides. The upregulation of this peroxidase activity by FSH could prevent atresia by protecting the membrane lipids and allowing differentiation of the follicle.

#### Structural molecule activity

We identified several interesting regulated genes in the structural molecule activity because they intervene in the extracellular matrix and cytoskeleton. Adhesion proteins, such as type IV collagen, increase the connections between cells and have also been shown to increase FSH receptors and progesterone production of GCs from immature porcine ovarian follicles [51]. The stimulation by FSH of *type IV collagen *gene expression could thus reinforce the effect of FSH and play a role in the local control of ovarian follicular dynamics [52].

## Conclusion

These results demonstrate the validity of both our cellular model and the SSH approach in identifying genes involved in response to FSH. In this way, and in addition to the regulation of the steroidogenesis and morphological changes already described, our data suggests that there is a role of FSH in the chromatin remodelling and protection against peroxides leading the follicle into a differentiation process rather than into atresia. Interestingly, we have been able to demonstrate the involvement and/or regulation of new genes such as *HP1-BP74*, *cox-2*, *CCT3, SCARB1*, *GPX3*, and also of unidentified genes. These new or unidentified genes will require further studies. Particularly, expression studies associated with histological techniques (*in situ *hybridization, immunohistochemistry) will allow a better understanding of the involvement of these genes.

SSH has demonstrated its efficiency in our hands. We will now use it to further improve our knowledge of folliculogenesis in pigs by analysing fresh GC from ovarian follicles at different developmental stages.

## Competing interests

The author(s) declare that they have no competing interests.

## Authors' contributions

AB conceived the design of the study, carried out the cell culture, mRNA extraction, regional assignment and gene ontology annotation, participated in the regulation study and drafted the manuscript. POF carried out the SSH, screening procedure, sequencing and regulation study. PD performed gene ontology annotation. GTK participated in its design and helped to write the manuscript. FH conceived of the study, supervised the experiment and helped to write the manuscript.

## Supplementary Material

Additional File 1 **Functional annotation data**. This table gives the results of the analysis for the first levels of the molecular function ontology tree. The regulated genes are listed according to their molecular function(s). These data are used to draw the donut chart (fig 3) Click here for file

## References

[B1] Otsuka F, Moore RK, Wang X, Sharma S, Miyoshi T, Shimasaki S (2005). Essential role of the oocyte in estrogen amplification of follicle-stimulating hormone signaling in granulosa cells. Endocrinology.

[B2] Kumar TR, Wang Y, Lu N, Matzuk MM (1997). Follicle stimulating hormone is required for ovarian follicle maturation but not male fertility. Nat Genet.

[B3] Plancha CE, Sanfins A, Rodrigues P, Albertini D (2005). Cell polarity during folliculogenesis and oogenesis. Reprod Biomed Online.

[B4] Tosser-Klopp G, Benne F, Bonnet A, Mulsant P, Gasser F, Hatey F (1997). A first catalog of genes involved in pig ovarian follicular differentiation. Mamm Genome.

[B5] Caetano AR, Johnson RK, Pomp D (2003). Generation and sequence characterization of a normalized cDNA library from swine ovarian follicles. Mamm Genome.

[B6] Caetano AR, Johnson RK, Ford JJ, Pomp D (2004). Microarray profiling for differential gene expression in ovaries and ovarian follicles of pigs selected for increased ovulation rate. Genetics.

[B7] Gladney CD, Bertani GR, Johnson RK, Pomp D (2004). Evaluation of gene expression in pigs selected for enhanced reproduction using differential display PCR and human microarrays: I. Ovarian follicles. J Anim Sci.

[B8] Diatchenko L, Lau YF, Campbell AP, Chenchik A, Moqadam F, Huang B, Lukyanov S, Lukyanov K, Gurskaya N, Sverdlov ED, Siebert PD (1996). Suppression subtractive hybridization: a method for generating differentially regulated or tissue-specific cDNA probes and libraries. Proc Natl Acad Sci U S A.

[B9] Hatey F, Langlois I, Mulsant P, Bonnet A, Benne F, Gasser F (1992). Gonadotropins induce accumulation of insulin-like growth factor I mRNA in pig granulosa cells in vitro. Mol Cell Endocrinol.

[B10] Hatey F, Gasparoux JP, Mulsant P, Bonnet A, Gasser F (1992). P450scc regulation in pig granulosa cells: Investigation into the mechanism of induction. J Steroid Biochem Mol Biol.

[B11] Chomczynski P, Sacchi N (1987). Single-step method of RNA isolation by acid guanidinium thiocyanate-phenol-chloroform extraction. Anal Biochem.

[B12] Hatey F, Mulsant P, Bonnet A, Benne F, Gasser F (1995). Protein kinase C inhibition of in vitro FSH-induced differentiation in pig granulosa cells. Mol Cell Endocrinol.

[B13] Tosser-Klopp G, Bonnet A, Yerle M, Hatey F (2001). Functional study and regional mapping of 44 hormono-regulated genes isolated from a porcine granulosa cell library. Genet Sel Evol.

[B14] SCHP.

[B15] Yerle M, Pinton P, Robic A, Alfonso A, Palvadeau Y, Delcros C, Hawken R, Alexander L, Beattie C, Schook L, Milan D, Gellin J (1998). Construction of a whole-genome radiation hybrid panel for high-resolution gene mapping in pigs. Cytogenet Cell Genet.

[B16] Lahbib-Mansais Y, Tosser-Klopp G, Leroux S, Cabau C, Karsenty E, Milan D, Barillot E, Yerle M, Hatey F, Gellin J (2003). Contribution to high-resolution mapping in pigs with 101 type I markers and progress in comparative map between humans and pigs. Mamm Genome.

[B17] IMpRH mapping tool.

[B18] Minegishi T, Hirakawa T, Abe K, Kishi H, Miyamoto K (2004). Effect of insulin-like growth factor-1 and 2,3,7,8-tetrachlorodibenzo-p-dioxin on the expression of luteinizing hormone receptors in cultured granulosa cells. Environ Sci.

[B19] Mao J, Smith MF, Rucker EB, Wu GM, McCauley TC, Cantley TC, Prather RS, Didion BA, Day BN (2004). Effect of epidermal growth factor and insulin-like growth factor I on porcine preantral follicular growth, antrum formation, and stimulation of granulosal cell proliferation and suppression of apoptosis in vitro. J Anim Sci.

[B20] May JV, Stephenson LA, Turzcynski CJ, Fong HW, Mau YH, Davis JS (1996). Transforming growth factor beta expression in the porcine ovary: evidence that theca cells are the major secretory source during antral follicle development. Biol Reprod.

[B21] Mulsant P, Lecerf F, Fabre S, Schibler L, Monget P, Lanneluc I, Pisselet C, Riquet J, Monniaux D, Callebaut I, Cribiu E, Thimonier J, Teyssier J, Bodin L, Cognie Y, Chitour N, Elsen JM (2001). Mutation in bone morphogenetic protein receptor-IB is associated with increased ovulation rate in Booroola Merino ewes. Proc Natl Acad Sci U S A.

[B22] Monget P, Fabre S, Mulsant P, Lecerf F, Elsen JM, Mazerbourg S, Pisselet C, Monniaux D (2002). Regulation of ovarian folliculogenesis by IGF and BMP system in domestic animals. Domest Anim Endocrinol.

[B23] McNatty KP, Juengel JL, Reader KL, Lun S, Myllymaa S, Lawrence SB, Western A, Meerasahib MF, Mottershead DG, Groome NP, Ritvos O, Laitinen MP (2005). Bone morphogenetic protein 15 and growth differentiation factor 9 co-operate to regulate granulosa cell function in ruminants. Reproduction.

[B24] Vitt UA, Hayashi M, Klein C, Hsueh AJ (2000). Growth differentiation factor-9 stimulates proliferation but suppresses the follicle-stimulating hormone-induced differentiation of cultured granulosa cells from small antral and preovulatory rat follicles. Biol Reprod.

[B25] Rockett JC, Swales KE, Esdaile DJ, Gibson GG (2000). Use of suppression-PCR subtractive hybridisation to identify genes that demonstrate altered expression in male rat and guinea pig livers following exposure to Wy-14,643, a peroxisome proliferator and non-genotoxic hepatocarcinogen. Toxicology.

[B26] Li WB, Gruber CE, Lin JJ, Lim R, D'Alessio JM, Jessee JA (1994). The isolation of differentially expressed genes in fibroblast growth factor stimulated BC3H1 cells by subtractive hybridization. Biotechniques.

[B27] Ben-Ze'ev A, Amsterdam A (1987). In vitro regulation of granulosa cell differentiation. Involvement of cytoskeletal protein expression. J Biol Chem.

[B28] Clouscard-Martinato C, Mulsant P, Robic A, Bonnet A, Gasser F, Hatey F (1998). Characterization of FSH-regulated genes isolated by mRNA differential display from pig ovarian granulosa cells. Anim Genet.

[B29] Goureau A, Yerle M, Schmitz A, Riquet J, Milan D, Pinton P, Frelat G, Gellin J (1996). Human and porcine correspondence of chromosome segments using bidirectional chromosome painting. Genomics.

[B30] Lahbib-Mansais Y, Dalias G, Milan D, Yerle M, Robic A, Gyapay G, Gellin J (1999). A successful strategy for comparative mapping with human ESTs: 65 new regional assignments in the pig. Mamm Genome.

[B31] Rohrer GA, Wise TH, Lunstra DD, Ford JJ (2001). Identification of genomic regions controlling plasma FSH concentrations in Meishan-White Composite boars. Physiol Genomics.

[B32] Gost.

[B33] Zehetner G (2003). OntoBlast function: From sequence similarities directly to potential functional annotations by ontology terms. Nucleic Acids Res.

[B34] Groth D, Lehrach H, Hennig S (2004). GOblet: a platform for Gene Ontology annotation of anonymous sequence data. Nucleic Acids Res.

[B35] Khan S, Situ G, Decker K, Schmidt CJ (2003). GoFigure: automated Gene Ontology annotation. Bioinformatics.

[B36] Conesa A, Gotz S, Garcia-Gomez JM, Terol J, Talon M, Robles M (2005). Blast2GO: a universal tool for annotation, visualization and analysis in functional genomics research. Bioinformatics.

[B37] Wang H, Ma WG, Tejada L, Zhang H, Morrow JD, Das SK, Dey SK (2004). Rescue of female infertility from the loss of cyclooxygenase-2 by compensatory up-regulation of cyclooxygenase-1 is a function of genetic makeup. J Biol Chem.

[B38] Wu YL, Wiltbank MC (2002). Transcriptional regulation of the cyclooxygenase-2 gene changes from protein kinase (PK) A- to PKC-dependence after luteinization of granulosa cells. Biol Reprod.

[B39] Elvin JA, Yan C, Matzuk MM (2000). Growth differentiation factor-9 stimulates progesterone synthesis in granulosa cells via a prostaglandin E2/EP2 receptor pathway. Proc Natl Acad Sci U S A.

[B40] Davis BJ, Lennard DE, Lee CA, Tiano HF, Morham SG, Wetsel WC, Langenbach R (1999). Anovulation in cyclooxygenase-2-deficient mice is restored by prostaglandin E2 and interleukin-1beta. Endocrinology.

[B41] Li Q, Li Z, Sun CX, Yu AC (2002). Identification of transcripts expressed under functional differentiation in primary culture of cerebral cortical neurons. Neurochem Res.

[B42] Soues S, Kann ML, Fouquet JP, Melki R (2003). The cytosolic chaperonin CCT associates to cytoplasmic microtubular structures during mammalian spermiogenesis and to heterochromatin in germline and somatic cells. Exp Cell Res.

[B43] Prange-Kiel J, Kreutzkamm C, Wehrenberg U, Rune GM (2001). Role of tumor necrosis factor in preovulatory follicles of swine. Biol Reprod.

[B44] Rosales-Torres AM, Avalos-Rodriguez A, Vergara-Onofre M, Hernandez-Perez O, Ballesteros LM, Garcia-Macedo R, Ortiz-Navarrete V, Rosado A (2000). Multiparametric study of atresia in ewe antral follicles: histology, flow cytometry, internucleosomal DNA fragmentation, and lysosomal enzyme activities in granulosa cells and follicular fluid. Mol Reprod Dev.

[B45] Dubois T, Mira JP, Feliers D, Solito E, Russo-Marie F, Oudinet JP (1998). Annexin V inhibits protein kinase C activity via a mechanism of phospholipid sequestration. Biochem J.

[B46] Belgrader P, Dey R, Berezney R (1991). Molecular cloning of matrin 3. A 125-kilodalton protein of the nuclear matrix contains an extensive acidic domain. J Biol Chem.

[B47] Xu TR, Rumsby MG (2004). Phorbol ester-induced translocation of PKC epsilon to the nucleus in fibroblasts: identification of nuclear PKC epsilon-associating proteins. FEBS Lett.

[B48] Svensson PA, Johnson MS, Ling C, Carlsson LM, Billig H, Carlsson B (1999). Scavenger receptor class B type I in the rat ovary: possible role in high density lipoprotein cholesterol uptake and in the recognition of apoptotic granulosa cells. Endocrinology.

[B49] Aitken RJ, Clarkson JS, Fishel S (1989). Generation of reactive oxygen species, lipid peroxidation, and human sperm function. Biol Reprod.

[B50] Foresta C, Flohe L, Garolla A, Roveri A, Ursini F, Maiorino M (2002). Male fertility is linked to the selenoprotein phospholipid hydroperoxide glutathione peroxidase. Biol Reprod.

[B51] Sites CK, Kessel B, LaBarbera AR (1996). Adhesion proteins increase cellular attachment, follicle-stimulating hormone receptors, and progesterone production in cultured porcine granulosa cells. Proc Soc Exp Biol Med.

[B52] Bortolussi M, Zanchetta R, Doliana R, Castellani I, Bressan GM, Lauria A (1989). Changes in the organization of the extracellular matrix in ovarian follicles during the preovulatory phase and atresia. An immunofluorescence study. Basic Appl Histochem.

