# Supplementary material for: Identification of differential gene expression in in vitro FSH treated pig granulosa cells using suppression subtractive hybridization
Source: Reprod Biol Endocrinol. 2006 Jul 7;4:35. doi: 10.1186/1477-7827-4-35 (PMC1533831; doi:10.1186/1477-7827-4-35)
Supplement: Additional File 1 — Functional annotation data. This table gives the results of the analysis for the first levels of the molecular function ontology tree. The regulated genes are listed according to their molecular function(s). These data are used to draw the donut chart (fig 3) [file 1477-7827-4-35-S1.pdf]

func	gene	prot_name	prot_def
antioxidant activity	H1-185	GPX3_HUMAN	Plasma glutathione peroxidase precursor
binding	H1-129	SCAP_HUMAN	Sterol regulatory element binding protein cleavage-activating protein
binding	H1-14	O62706	Gag protein
binding	H1-185	GPX3_HUMAN	Plasma glutathione peroxidase precursor
binding	H1-2	AKC2_HUMAN	Aldo-keto reductase family 1 member C2
binding	H1-280	C11A_PIG	Cytochrome P450 11A1, mitochondrial precursor
binding	H1-357	TCPG_HUMAN	T-complex protein 1, gamma subunit
binding	H1-94	TENA_PIG	Tenascin precursor
binding	H2-105	Q9UHY0	hp1-bp74 protein
binding	H2-108	COX2_PIG	Cytochrome c oxidase polypeptide II
binding	H2-146	ACTA_HUMAN	Actin, aortic smooth muscle
binding	H2-175	MAT3_HUMAN	Matrin 3
binding	H2-222	ANX5_HUMAN	Annexin A5
binding	H2-265	INPP_BOVIN	Inositol polyphosphate 1-phosphatase
binding	H2-44	TSP1_BOVIN	Thrombospondin 1 precursor
binding	H2-96	Q9QZ83	Gamma actin-like protein
catalytic activity	H1-1	Q9N119	3-beta-hydroxysteroid dehydrogenase/delta-5-delta-4 isomerase
catalytic activity	H1-185	GPX3_HUMAN	Plasma glutathione peroxidase precursor
catalytic activity	H1-2	AKC2_HUMAN	Aldo-keto reductase family 1 member C2
catalytic activity	H1-280	C11A_PIG	Cytochrome P450 11A1, mitochondrial precursor
catalytic activity	H2-108	COX2_PIG	Cytochrome c oxidase polypeptide II
catalytic activity	H2-265	INPP_BOVIN	Inositol polyphosphate 1-phosphatase
enzyme regulator activity	H2-222	ANX5_HUMAN	Annexin A5
signal transducer activity	H1-90	SRC1_PIG	Scavenger receptor class B member 1
structural molecule activity	H1-14	O62706	Gag protein
structural molecule activity	H1-61	CA54_HUMAN	Collagen alpha 5(IV) chain precursor

structural molecule activity	H2-146	ACTA_HUMAN	Actin, aortic smooth muscle
structural molecule activity	H2-175	MAT3_HUMAN	Matrin 3
structural molecule activity	H2-44	TSP1_BOVIN	Thrombospondin 1 precursor
structural molecule activity	H2-96	Q9QZ83	Gamma actin-like protein
transporter activity	H1-2	AKC2_HUMAN	Aldo-keto reductase family 1 member C2
transporter activity	H2-108	COX2_PIG	Cytochrome c oxidase polypeptide II
unclassified	H1-180		
unclassified	H1-246		
unclassified	H1-276		
unclassified	H1-294		
unclassified	H2-170		
unclassified	H2-92		
unclassified	H2-95		
